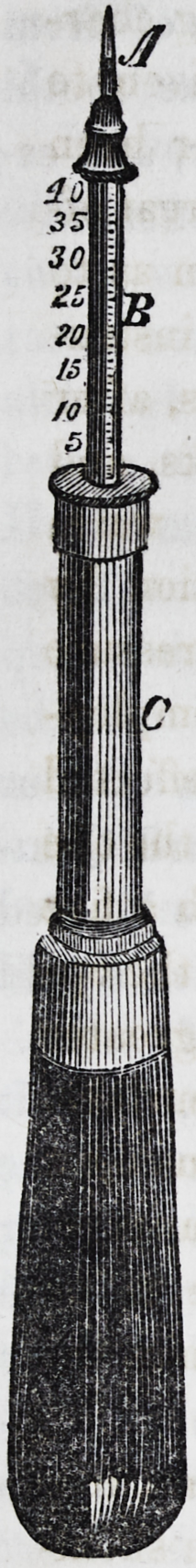# Dynamometer

**Published:** 1856-04

**Authors:** 


### ARTICLE XIX.

### *Dynamometer.*

The instrument which we have been using for the purpose of experimenting on the subject of the condensation of gold foil and crystal gold, is made by extending a beam or shaft through a common spring balance of the stores, which draws fifty pounds. This shaft is fastened to the spring instead of the wire, which is used in these balances. The shaft is left extended above the plate of the instrument sufficiently to allow of free motion, and on the upper extremity there is a small vice firmly attached, and into which a small iron ingot can be fastened at pleasure. This shaft is held in its position, when pressure is made, by a plate through which it passes at either end of the scale-plate of this common balance, and instead of its being pulled at, as in weighing articles by the shopkeeper, it is pushed upon from the utter extremity, and, of course, registers the weight on the old scale-plate; the difference is, therefore, only between pushing and pulling. Near the ingot is placed a graduating rest for the the thumb or finger of the operator, so that he can operate on the ingot, and imitate nearly all the positions assumed while operating on the mouth—thus testing very well the amount of force he is capable of exerting in those different attitudes of the hand, except that he can effect much more pressure on the instrument than upon the mouth, which we will hereafter show. It is believed by many operators that as much force can be applied in plugging teeth with the instrument held between the fingers as if it be grasped in the hand. This is not so. The greatest amount that we can apply on the dynamometer, with the instrument in the fingers, is from twelve to eighteen pounds; while, if we grasp the instrument in the hand, and let the end of the handle rest against the palm, near the wrist, we can, with much less bodily effort, exert fifty pounds, and this relative proportion is kept up when operating on the mouth. It is true that a good plug can be made by using the instrument in that way, but it is at a much greater expenditure of labor and time than if the instrument be larger and held in the hand. In operating upon the lateral surfaces of teeth, there is seldom a greater force exerted than from five to ten pounds; that is, direct forward force. The amount of force lost by the yielding character of the parts operated upon, and lateral support given to the instrument in precaution against slipping, has never been estimated by the operator as lost: in other words, the great efforts of the operator are so distributed as to deceive him as to the amount transmitted to the condensing point of the instrument. The many complaints of the broken-down dentists, after a few years' constant and hard operating, and the weeks, and even months, of relaxation taken by some of them in warm weather, makes this a matter worthy of serious reflection by every member of the profession. A certain amount of pressure is absolutely essential for condensing gold, and must be employed by one as well as the other, and if only five pounds is effected by the one, and ten by the other, it counts in time against the one only employing five; and again, a plug that is made with a five or ten pounds pressure, and feels to be condensed under the operator's instrument, will very readily be crushed by a greater pressure with an instrument with the same condensing surface. The great length of time consumed in properly condensing a plug with a small instrument, and used under disadvantage, makes the operation of plugging a tooth too expensive to be within the reach of many persons to pay; besides, it limits the operator to a very few operations per day.

The dentist should learn, as one of the most important features in his *skill* in plugging teeth, to operate from the shoulder, and the instrument grasped full in the hand, as all the strength used to hold on to it, to prevent it slipping through his fingers, is lost to its condensing point and to the shoulder. We know a number of operators who fully appreciate the necessity of a proper condensation of a plug, but exhaust their strength in doing it. This should not be. We have also another instrument for testing the amount of pressure that is usually made in operating on the mouth, and which can be applied to crown cavities in the superior molars. It is about nine inches long, and can be understood by referring to the accompanying cut, which is reduced to about half size.

*A* is the condensing point or bit, in a socket, so that any size may be used. *B* is the shaft, and on which the scale is marked, to register the amount of force applied to the instrument. *C* is a hollow cylinder in which the spring is contained. When the extremity of the shaft *B* is depressed, the opposite extremity runs into the handle *D*, which is hollow to receive it. It is with this instrument that we have corrected the very erroneous idea we entertained heretofore with regard to the amount of direct pressure we supposed we were in the habit of exerting upon a plug in the mouth, as well as the amount of pressure different patients and teeth can bear. It is a difficult matter for us to apply more than ten or twelve pounds of pressure on a superior molar of a patient of that many years of age, or a nervous and yielding patient. We never used two hundred pounds and over, in these delicate cases; but when we have an older patient, or a hard head and stiff neck, and a molar well set in a well developed jaw, and the patient firmly seated in the chair, we can apply as much as twenty-five, and even in some cases thirty pounds: this we venture when the plug is nearly done, otherwise we would fear thrusting the plugger through the tooth. A friend of ours said he thought he was in the habit of applying from sixty to eighty pounds pressure. We sent him the instrument; he applied it to a plug in the crown of a superior molar, and at twenty-five pounds the point of the instrument penetrated the plug, as he said, about the thirtieth of an inch. In experimenting on plugs out of the mouth, in teeth that we have extracted, we have not found any plugs that would bear thirty pounds, very few more than fifteen, some not ten; in fact, every plug that we have tried were at best mere sponges, comparatively. Many of those plugs, when made and dry, would doubtless have borne a much greater pressure without giving way, but such is the influence of moisture, or something else, that they are easily penetrated by the instrument after they are worn some time. It is extremely exhausting for an operator to keep up a prolonged pressure of from fifteen to twenty pounds upon a crown plug of a molar tooth. We will continue our article in the next News Letter, and especially on the *cylindrically* prepared gold.—*News Letter.*

## Figures and Tables

**Figure f1:**